# Draft Genome Sequence of Enterobacter kobei M4-VN, Isolated from Potatoes with Soft Rot Disease

**DOI:** 10.1128/MRA.00908-20

**Published:** 2020-09-03

**Authors:** Nguyen Cong Thanh, Yasuhiro Fujino, Yasuaki Hiromasa, Katsumi Doi

**Affiliations:** a Microbial Genetic Division, Institute of Genetic Resources, Faculty of Agriculture, Kyushu University, Fukuoka, Japan; b Plant Protection Research Institute, Hanoi, Vietnam; c Attached Promotive Center for International Education and Research of Agriculture, Faculty of Agriculture, Kyushu University, Fukuoka, Japan; Indiana University, Bloomington

## Abstract

Enterobacter kobei M4-VN, isolated from potatoes with soft rot disease in Vietnam, contains a total of 4,754,309 bp with 4,424 predicted coding sequences and a G+C content of 55.1%.

## ANNOUNCEMENT

*Enterobacter* species are Gram-negative facultatively anaerobic bacteria belonging to the family *Enterobacteriaceae* (1, 2). Mostly, the species have been reported to be nosocomial pathogens showing resistance to disinfectants and antimicrobial chemicals (3). However, some of them have been reported as phytopathogens, such as Enterobacter asburiae isolated from konjac (Amorphophallus konjac) in China and Enterobacter cloacae isolated from chili pepper (Capsicum annuum L.) and dragon fruit (*Hylocereus* spp.) (4–7). Identification of the order *Enterobacteriales* has been difficult with the 16S rRNA gene approach and other single-gene and limited multigene approaches (2). Therefore, sequencing the whole genome might be useful for species differentiation and prediction of pathogenicity (8).

The strain used in this study (M4-VN) was isolated from potatoes with soft rot disease in Hanoi, Vietnam. The potatoes were washed with sterile water and 70% alcohol to remove surface contaminants, rinsed with sterile distilled water, and cut into specimens. The specimens that had the disease were selected and streaked onto lysogeny broth (LB) plates and inoculated at 37°C for 24 to 48 h. The bacterial colonies were purified with serial streaking.

A single colony was then cultivated anaerobically overnight at 37°C in LB. Genomic DNA was then extracted and purified as described by Marmur (9), with some modifications. A sequencing library was prepared for the HiSeq platform (Illumina, San Diego, CA, USA) with the VAHTS universal DNA library prep kit, following the manufacturer’s instructions. The resulting library was sequenced on an Illumina HiSeq platform using a 2 × 150-bp paired-end configuration, generating a total of 14,421,674 raw reads (Genewiz, China). Low-quality read filtering was performed using Cutadapt ver. 1.9.1 (10). *De novo* assembly was performed with KmerGenie (ver. 1.6982), Velvet (ver. 1.2.10), SSPACE (ver. 3.0), and GapFiller (ver. 1-10) (11–15). Annotation was performed using the DFAST pipeline (16, 17). Determination of closely related strains was performed using the TYGS platform (18).

The assembled genome of *Enterobacter* sp. strain M4-VN contained 18 contigs with a total of 4,754,309 bp (G+C content, 55.1%), an *N*_50_ value of 636,975 bp, an average sequencing depth of 449.97, and minimum and maximum contig lengths of 1,812 and 949,261 bp, respectively. Annotation revealed 4,424 predicted coding regions, 65 tRNA genes, 7 rRNA genes, and 1 CRISPR gene. From the annotation, we identified a gene encoding pectinesterase (19, 20), the TonB protein, 4 genes encoding the TonB-dependent receptor, a gene encoding the M16 proteases (*fusC*) (21), and genes related to susceptibility to antibacterial plant chemicals (*tolC*) (22). The results of digital DNA-DNA hybridization (dDDH) showed that strain M4-VN is homologous (92.6%) with Enterobacter kobei DSM 13645^T^. The percentage is remarkably higher than the second closest dDDH values (42.5%) calculated with Enterobacter bugandensis EB-247^T^ and Enterobacter chengduensis WCHECl-C4^T^. This suggests that strain M4-VN belongs to Enterobacter kobei.

### Data availability.

The genome sequence and annotation data for Enterobacter kobei M4-VN were deposited in DDBJ/GenBank under BioProject number PRJDB9609, BioSample number SAMD00218344, DRA number DRA010546, and the accession numbers BLVN01000001 through BLVN01000018.

## References

[1] Brenner DJ, Farmer JJ III 2005 Enterobacteriales, p 587–850. *In* Garrity G, Brenner DJ, Krieg NR, Staley JR (ed), Bergey’s Manual of Systematic Bacteriology, vol 2 Springer-Verlag, New York, NY.

[2] Adeolu M, Alnajar S, Naushad S, Gupta RS 2016 Genome-based phylogeny and taxonomy of the “*Enterobacteriales*”: proposal for *Enterobacterales* ord. nov. divided into the families *Enterobacteriaceae*, *Erwiniaceae* fam. nov., *Pectobacteriaceae* fam. nov., *Yersiniaceae* fam. nov., *Hafniaceae* fam. nov., *Morganellaceae* fam. nov., and *Budviciaceae* fam. nov. Int J Syst Evol Microbiol 66:5575–5599. doi:10.1099/ijsem.0.001485. 27620848

[3] Martin-Carnahan A, Joseph SW 2005 Aeromonadales ord. nov, p 556–587. *In* Garrity G, Brenner DJ, Krieg NR, Staley JR (ed), Bergey’s Manual of Systematic Bacteriology, vol 2 Springer-Verlag, New York, NY.

[4] Wang G-F, Xie G-L, Zhu B, Huang J-S, Liu B, Kawicha P, Benyon L, Duan Y-P 2010 Identification and characterization of the *Enterobacter* complex causing mulberry (*Morus alba*) wilt disease in China. Eur J Plant Pathol 126:465–478. doi:10.1007/s10658-009-9552-x.

[5] Wu J, Ding Z, Diao Y, Hu Z 2011 First report on *Enterobacter* sp. causing soft rot of *Amorphophallus konjac* in China. J Gen Plant Pathol 77:312–314. doi:10.1007/s10327-011-0330-1.

[6] García-González T, Sáenz-Hidalgo HK, Silva-Rojas HV, Morales-Nieto C, Vancheva T, Koebnik R, Ávila-Quezada GD 2018 *Enterobacter cloacae*, an emerging plant-pathogenic bacterium affecting chili pepper seedlings. Plant Pathol J 34:1–10. doi:10.5423/PPJ.OA.06.2017.0128. 29422783 PMC5796745

[7] Masyahit M, Sijam K, Awang Y, Ghazali MSM 2009 First report on bacterial soft rot disease on dragon fruit (*Hylocereus* spp.) caused by *Enterobacter cloacae* in Peninsular Malaysia. Int J Agric Biol 11:659–666.

[8] Naum M, Brown EW, Mason-Gamer RJ 2008 Is 16S rDNA a reliable phylogenetic marker to characterize relationships below the family level in the *Enterobacteriaceae*? J Mol Evol 66:630–642. doi:10.1007/s00239-008-9115-3. 18504519

[9] Marmur J 1961 A procedure for the isolation of deoxyribonucleic acid from micro-organisms. J Mol Biol 3:208–218. doi:10.1016/S0022-2836(61)80047-8.

[10] Martin M 2011 Cutadapt removes adapter sequences from high-throughput sequencing reads. EMBnet J 17:10. doi:10.14806/ej.17.1.200.

[11] Zerbino DR, Birney E 2008 Velvet: algorithms for de novo short read assembly using de Bruijn graphs. Genome Res 18:821–829. doi:10.1101/gr.074492.107. 18349386 PMC2336801

[12] Boetzer M, Henkel CV, Jansen HJ, Butler D, Pirovano W 2011 Scaffolding pre-assembled contigs using SSPACE. Bioinformatics 27:578–579. doi:10.1093/bioinformatics/btq683. 21149342

[13] Boetzer M, Pirovano W 2012 Toward almost closed genomes with GapFiller. Genome Biol 13:R56. doi:10.1186/gb-2012-13-6-r56. 22731987 PMC3446322

[14] Zerbino DR, McEwen GK, Margulies EH, Birney E 2009 Pebble and Rock Band: heuristic resolution of repeats and scaffolding in the Velvet short-read de novo assembler. PLoS One 4:e8407. doi:10.1371/journal.pone.0008407. 20027311 PMC2793427

[15] Chikhi R, Medvedev P 2014 Informed and automated k-mer size selection for genome assembly. Bioinformatics 30:31–37. doi:10.1093/bioinformatics/btt310. 23732276

[16] Tanizawa Y, Fujisawa T, Kaminuma E, Nakamura Y, Arita M 2016 DFAST and DAGA: Web-based integrated genome annotation tools and resources. Biosci Microbiota Food Health 35:173–184. doi:10.12938/bmfh.16-003. 27867804 PMC5107635

[17] Tanizawa Y, Fujisawa T, Nakamura Y 2018 DFAST: a flexible prokaryotic genome annotation pipeline for faster genome publication. Bioinformatics 34:1037–1039. doi:10.1093/bioinformatics/btx713. 29106469 PMC5860143

[18] Meier-Kolthoff JP, Göker M 2019 TYGS is an automated high-throughput platform for state-of-the-art genome-based taxonomy. Nat Commun 10:2182. doi:10.1038/s41467-019-10210-3. 31097708 PMC6522516

[19] Gainvors A, Frézier V, Lemaresquier H, Lequart C, Aigle M, Belarbi A 1994 Detection of polygalacturonase, pectin‐lyase and pectin‐esterase activities in a *Saccharomyces cerevisiae* strain. Yeast 10:1311–1319. doi:10.1002/yea.320101008. 7900420

[20] Gavrilovic V, Obradovic A, Arsenijevic M 2001 Bacterial soft rot of carrot, parsley and celery, p 269*–*271. *In* De Boer SH (ed), Plant pathogenic bacteria. Proceedings of the 10th International Conference on Plant Pathogenic Bacteria, Charlottetown, PEI, Canada.

[21] Mosbahi K, Wojnowska M, Albalat A, Walker D 2018 Bacterial iron acquisition mediated by outer membrane translocation and cleavage of a host protein. Proc Natl Acad Sci U S A 115:6840–6845. doi:10.1073/pnas.1800672115. 29891657 PMC6042079

[22] Lee DH, Lim J-A, Lee J, Roh E, Jung K, Choi M, Oh C, Ryu S, Yun J, Heu S 2013 Characterization of genes required for the pathogenicity of *Pectobacterium carotovorum* subsp. *carotovorum* Pcc21 in Chinese cabbage. Microbiology 159:1487–1496. doi:10.1099/mic.0.067280-0. 23676432 PMC3749726

